# Evaluating Spatial Overlap and Relatedness of White-tailed Deer in a Chronic Wasting Disease Management Zone

**DOI:** 10.1371/journal.pone.0056568

**Published:** 2013-02-20

**Authors:** Seth B. Magle, Michael D. Samuel, Timothy R. Van Deelen, Stacie J. Robinson, Nancy E. Mathews

**Affiliations:** 1 Urban Wildlife Institute, Lincoln Park Zoo, Chicago, Illinois, United States of America; 2 U.S. Geological Survey, Wisconsin Cooperative Wildlife Research Unit, University of Wisconsin, Madison, Wisconsin, United States of America; 3 Department of Forest and Wildlife Ecology, University of Wisconsin, Madison, Wisconsin, United States of America; 4 Nelson Institute for Environmental Studies, University of Wisconsin, Madison, Wisconsin, United States of America; University of Georgia, United States of America

## Abstract

Wildlife disease transmission, at a local scale, can occur from interactions between infected and susceptible conspecifics or from a contaminated environment. Thus, the degree of spatial overlap and rate of contact among deer is likely to impact both direct and indirect transmission of infectious diseases such chronic wasting disease (CWD) or bovine tuberculosis. We identified a strong relationship between degree of spatial overlap (volume of intersection) and genetic relatedness for female white-tailed deer in Wisconsin’s area of highest CWD prevalence. We used volume of intersection as a surrogate for contact rates between deer and concluded that related deer are more likely to have contact, which may drive disease transmission dynamics. In addition, we found that age of deer influences overlap, with fawns exhibiting the highest degree of overlap with other deer. Our results further support the finding that female social groups have higher contact among related deer which can result in transmission of infectious diseases. We suggest that control of large social groups comprised of closely related deer may be an effective strategy in slowing the transmission of infectious pathogens, and CWD in particular.

## Introduction

Social organization and interactions among individuals play an important role in the transmission and potential management of infectious wildlife diseases [1], [2]. Many host characteristics such as sex, age, relatedness, density, social group composition, inter-group movement and isolation can influence the duration and intensity of contacts and disease transmission [1], [3]. Understanding how contact rates and social organization influences disease transmission and spread is a challenging issue in disease ecology yet it is critical for disease management [2], [4]. Complex social behaviors are typical for wild mammals and can result in disease transmission rates that are not explained by density alone [1], [2]. In social species with stable group membership, given that at least one member is infected, individuals from the same group may have a higher rate of infection than non-members. White-tailed deer (*Odocoileus virginianus*) are one such social species known to associate in stable matrilineal groups [5], [6], [7]. Female white-tailed deer generally associate more closely with relatives than with non-relatives [8], [9], [10]. Although the nature and persistence of these interactions are still under study [3], [6], [7], this general pattern has widespread acceptance and is sometimes referred to as the “Rose-petal theory” [11]. However, deer social behavior, site fidelity, and spatial overlap can vary among different habitats deer density, and hunting pressure [12], [13], [14], [15], [16].

Chronic wasting disease (CWD) is a fatal neurodegenerative disease, posing serious and complex challenges for deer management [17], [18]. In captive studies, CWD can be transmitted through animal-to-animal contact and indirect environmental contamination [19], [20]. The relative importance of these transmission routes is not known in free-ranging deer. Probability of CWD infection in harvested female deer was recently found to be strongly influenced by genetic relatedness and, only incidentally, by spatial proximity to other infected females [21]. Ultimately, local transmission of CWD results from individual deer movements that lead to interactions with conspecifics or the environment. In particular, the degree of spatial overlap and contact among deer is likely to impact both direct and indirect transmission of CWD, and other infectious diseases such as bovine tuberculosis [3], [6], [22].

Although infection patterns and spatial distribution of CWD in Wisconsin have recently been described, there is limited empirical information on white-tailed deer behavior and interaction related to potential disease transmission and spread. Following discovery of the disease, an important management goal of the Wisconsin Department of Natural Resources (WDNR) was to reduce the deer population in the area where disease prevalence was highest, termed the Disease Eradication Zone (DEZ, [23]). Population reductions are most likely to be effective in reducing disease prevalence if disease transmission is density-dependent. Information on deer movement, space use, social structure and potential interaction are necessary to understand local-scale infectious contacts that generate the emergent dynamics of disease transmission on the landscape.

Although previous studies on deer spatial overlap have been conducted [3], [6], we provide the first study evaluating links between deer spatial overlap, measured using VHF telemetry data, and deer relatedness based on microsatellite genetic markers. Based on female social structure, we hypothesize that related deer have greater spatial overlap, and thus more direct contact, than unrelated deer in the same areas. We also hypothesize that fawns, who are dependent on their mothers, will overlap with adjacent deer more strongly than yearlings or adults. These overlap areas, where multiple deer share space, are the most likely regions for either direct or indirect transmission of CWD [3]. As such, understanding the factors that determine the degree of overlap, and consequently the amount of direct contact, between deer will be critical to understanding and mitigating the spread of CWD and other infectious diseases.

## Methods

### Deer Tracking

Our study was conducted within two areas of Wisconsin’s Disease Eradication Zone (DEZ; see [24], [25]), between January and April, 2003–2008. We captured 173 individual white-tailed deer (113 females, 60 males; [24], [25], using modified Clover and Stephenson box traps, rocket nets [26], drop-nets [27], and darting. We aged deer as fawns (<1 year), yearlings (≥1 year, <2 years), and adults (≥2 years) by tooth wear and replacement [28]. We chemically immobilized captured deer [25] and affixed VHF radio collars. We tested deer for CWD using tonsillar biopsy [29] and collected blood and tissue samples for genetic analysis. Five deer initially tested CWD-positive (4 females, 1 male) and were culled.

We triangulated locations of radio-collared deer using 3 to 5 azimuths collected from fixed telemetry stations and obtained locations on a 24 hr basis, using rotating start times. We located radio-collared deer roughly 3 times/wk from 2003 to 2008 (Range: 1–6, Mean: 3.2). We estimated locations using Location of a Signal (LOAS), Version 2.09 [30], for groups of azimuths obtained within 20 min of each other. Estimates of positional error were ≤0.05 km^2^. We spaced relocations of individual deer ≥6 hrs apart to minimize temporal autocorrelation.

### Ethics Statement

The University of Wisconsin-Madison (UW) College of Agriculture and Life Sciences’ Animal Care and Use Committee (ACUC, Permit No. A-3368-01), UW Research Animal Resources Center (Permit No. A01088309-02), and the Wisconsin Department of Natural Resources (WDNR, Scientific Collector’s permit No. SCP-SCR-018-0202) approved capture and handling methods. Landowner permissions were acquired for capture on private lands.

### Inclusion Criteria

We selected a subset of radio-tracked deer for analysis in this study. We excluded all male deer older than 1 yr from analysis because they display less philopatry than females and often engage in long-range movements [9]. Male fawns were retained due to their close association with their mother prior to dispersal. In addition, to limit the number of possible deer-pairs with extremely low or no overlap, we used only deer-pairs trapped within 1.5 km of each other. To address the potential lack of spatial independence among deer-pairs, we assigned deer trapped within 1.5 km of one another to one of 7 distinct capture groups which were treated as a random effect in our analysis. We used only deer with >50 estimated locations (n = 105) during a given year.

### Overlap Modeling

We used volume of intersection (VI) of utilization distributions [6], [31] as our measure of spatial overlap. This measure has previously been found to correlate strongly with contact rates in deer [6]. Utilization distributions are three-dimensional probability densities that indicate relative space use based on point locations [32], [33]. The VI is the approximate spatial integral of the square root of the product of two fixed utilization distribution kernels. VI values range from 0 to 1, with 0 representing no overlap, and 1 indicating complete overlap. For included deer-pairs, VI values were calculated for all points collected within one year (years were defined to begin on 10 May). To avoid correlation from multiple observations of the same deer-pairs (example: Deer 1003 and 1004 in years 2003, 2004, and 2005), we selected a single VI calculated for each deer-pair, from the year with the most combined locations. Volumes of intersection (VI) were then logit-transformed to facilitate analysis using linear models.

### Relatedness

Whole genomic DNA was extracted from deer samples using a Qiagen DNeasy extraction kit (Qiagen Inc., Valencia, CA) following the manufacturer’s protocol for either 100 ul of blood or 20 mg of tissue from ear punch samples (all samples frozen since collection). We amplified 13 highly variable microsatellite loci using PCR with the Qiagen multiplex PCR kit [34]. We re-genotyped 32 individuals to assess errors in genotyping. We calculated Hardy-Weinberg equilibrium (HWE) and expected versus observed numbers of heterozygotes and homozygotes for all loci (using Genepop on the web; [35]) to assess data quality and assumptions for population genetics models. We used probability of identity statistics (PID and PIDsibs, performed in GenAlEx, [36]) to ensure adequate power to identify closely related individuals in our dataset. In order to provide a more genetically representative background with which to test our hypotheses, we supplemented the current sample with 100 additional deer from the same general geographic area that were genotyped in a collaborating study (analyzed using the same genetic methods [21]).

We calculated genetic relatedness and pedigree relationships using maximum likelihood [37] methods in program ML-Relate [38]. Pair-wise genetic relatedness (R_xy_) ranges from 0 to 1 representing the proportion of allelic composition shared between individuals x and y [39]. Theory suggests first-order relatives (full siblings or parent-offspring pairs) should share half their genetic makeup (i.e., R_xy_ = 0.5). Half siblings or grandparent-grandchild pairs, termed second-order relatives, would be expected to share only a quarter of their ancestry (i.e., R_xy_ = 0.25). We evaluated the importance of relationship classes, in addition to continuous R_xy_ values, using three classes of relatedness: first order kin (R_xy_ of 0.51–1), second order kin (0.26–0.5), and unrelated (0–0.25).

### Age and Sex

Age and sex categories were male fawn (B), female fawn (G), yearling female (Y), and adult female (A). Each deer-pair was assigned an age-sex class category corresponding to the ages of each deer in the pair, for example; ‘AA’ for two adult females, and ‘BY’ for a male fawn-female yearling pair. We also created an alternate age-class variable consisting of only fawns (F) regardless of sex, yearling females, and adult females. We used May 10 to define a new year in the analysis, at which point fawns were transferred to the yearling class, and yearlings to the adult class.

### Statistical Analyses

Overlap values derived from logit-transformed VIs were related to predictor variables including relatedness (continuous R_xy_ values and kinship categories) and age-sex classes using linear mixed effects models and maximum likelihood estimation (MLE) [40] with the nlme package in program R [41]. Fixed effects included relatedness and deer-pair age, while random effects were deer-pair nested within capture group (all deer captured within 1500 m). We used random effects for capture group and deer-pair to account for potential spatial autocorrelation and lack of independence for deer with multiple pairs, respectively. We first fit a global model using restricted maximum likelihood (REML), and tested the importance of the random effects using likelihood ratio tests [42]. We then recomputed our models using maximum likelihood (ML) to test the importance of fixed effects, with models selection based on Akaike’s information criterion adjusted for small sample sizes (AICc, [43]) and AIC weights. We used odds ratios to determine effect sizes for predictor variables.

Initial models failed to converge due to very low representation of some age class-capture group combinations. Because of this, we created two subsets of the data in which all age-sex class-capture group combinations contained sufficient data. The first, dataset_adult_, included only those deer-pairs with at least 1 adult, excluding deer-pair age classes such as fawn-yearling and fawn-fawn, which were absent in some capture groups. However, it does include representation from all 7 capture groups. The second, dataset_capgroup_, included only deer from capture groups 1 and 2, which contained the majority (70.4%) of all deer-pairs, including at least 27 deer in each age-pair type.

## Results

Over the 13 loci, no errors were found in the genotyping of the 32 repeated individuals. No deviations from Hardy-Weinberg equilibrium were found after Bonferonni correction for multiple loci tests. The locus set was highly variable, yielding sufficient power to distinguish among closely related individuals (PID = 7.18E-18, and PIDsibs = 1.30E-06).

Dataset_adult_ consisted of 668 deer-pairs with at least one adult. Dataset_capgroup_ consisted of 615 deer-pairs from capture groups 1 and 2. We tested a sequence of age and R_xy_ models, based on a priori knowledge of deer biology, for each of our two datasets (Table 1). Likelihood ratio tests applied to our global model indicated that both capture group and deer-pair (nested within capture group) contained significant explanatory power as random effects (capture group χ^2^ = 4.29, p = 0.04; deer-pair χ^2^ = 23.84, p<0.001) so both were used in all subsequent models.

**Table 1:** Results of AIC model selection procedure to determine the best models predicting white-tailed deer spatial overlap in Wisconsin.

Dataset	Variables			K	Delta AICc	Weight
1 (Pairs with at least one adult)	Age classes	Relatedness (Rxy-cat)		4	0.00	0.71
	Age classes (sex effects- fawns)	Relatedness (Rxy-cat)		4	1.95	0.27
	Age classes	Relatedness (Rxy)		4	7.66	0.02
	Age classes (sex effects- fawns)	Relatedness (Rxy)		4	9.59	0.01
	Age classes			3	13.63	<0.01
	Relatedness (Rxy- cat)			3	15.22	<0.01
	Age classes (sex effects- fawns)			3	15.55	<0.01
	Relatedness (Rxy)			3	24.62	<0.01
2 (Capture groups 1 and 2)	Age classes	Relatedness (Rxy- cat)		4	0.00	0.88
	Age classes	Relatedness (Rxy)		4	4.69	0.08
	Age classes (sex effects- fawns)	Relatedness (Rxy- cat)		4	7.38	0.02
	Age classes			3	8.05	0.02
	Age classes (sex effects- fawns)	Relatedness (Rxy)		4	12.23	<0.01
	Relatedness (Rxy- cat)			3	13.93	<0.01
	Age classes (sex effects- fawns)			3	15.53	<0.01
	Relatedness (Rxy)			3	19.74	<0.01

Dataset_adult_ contains only deer-pairs including at least one adult deer, and dataset_capgroup_ contains only capture groups 1 and 2 (see text). All models contain capture group and deer-pair as random effects. Age classes are either in categories of adult, yearling, and fawn (if lacking sex effects), or adult, yearling, male fawn, and female fawn. Relatedness is either represented as a continuous variable (R_xy_) or categorical (R_xy-cat_), in 3 categories consisting of R_xy_ of 0–0.25, 0.26–0.5, and 0.5–1.

**Figure 1:**
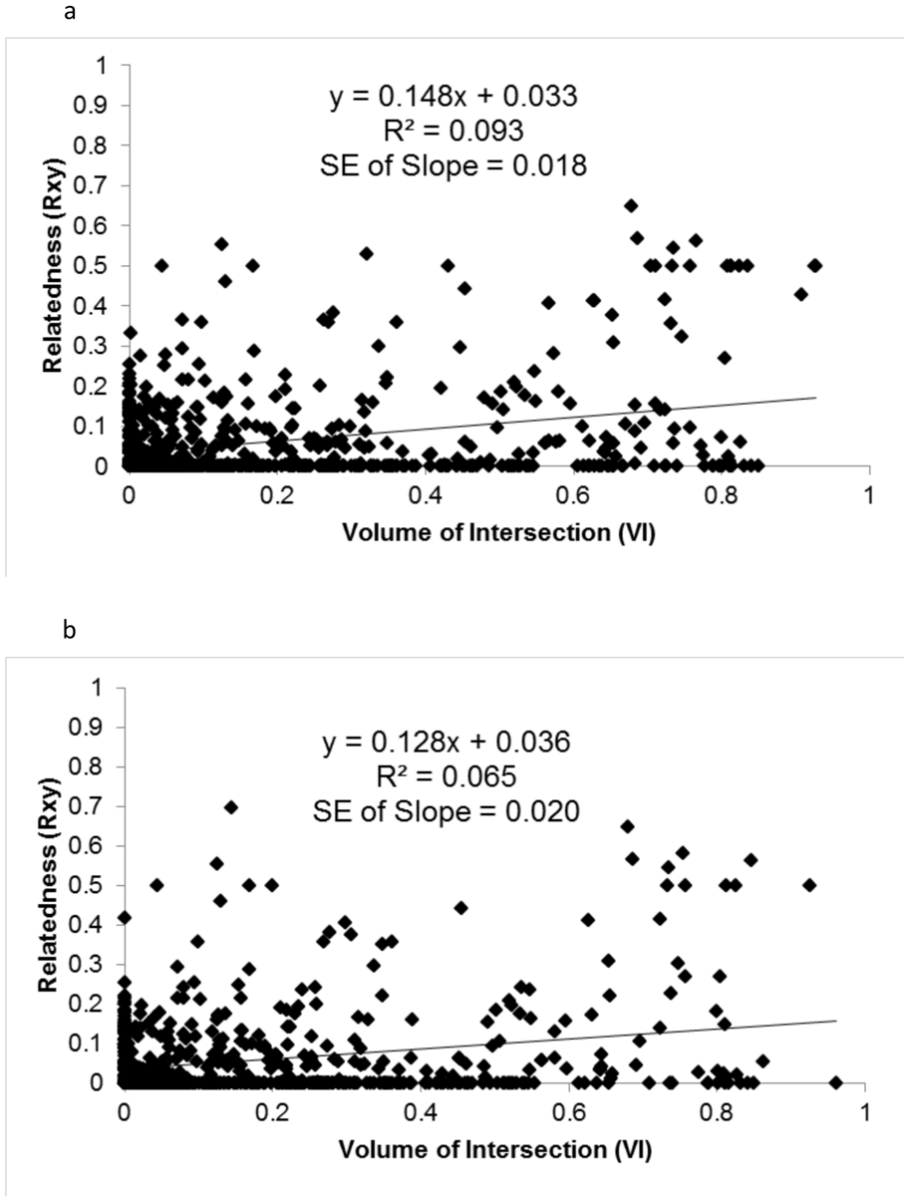
Scatterplots showing the relationship between degree of overlap and relatedness for white-tailed deer-pairs. Figure 1a is generated from the dataset where each pair contains at least one adult (dataset_adult_), and figure 1b is generated from the dataset using only deer-pairs from capture groups 1 and 2 (dataset_capgroup_). Figure 1a. Figure 1b.

**Figure 2:**
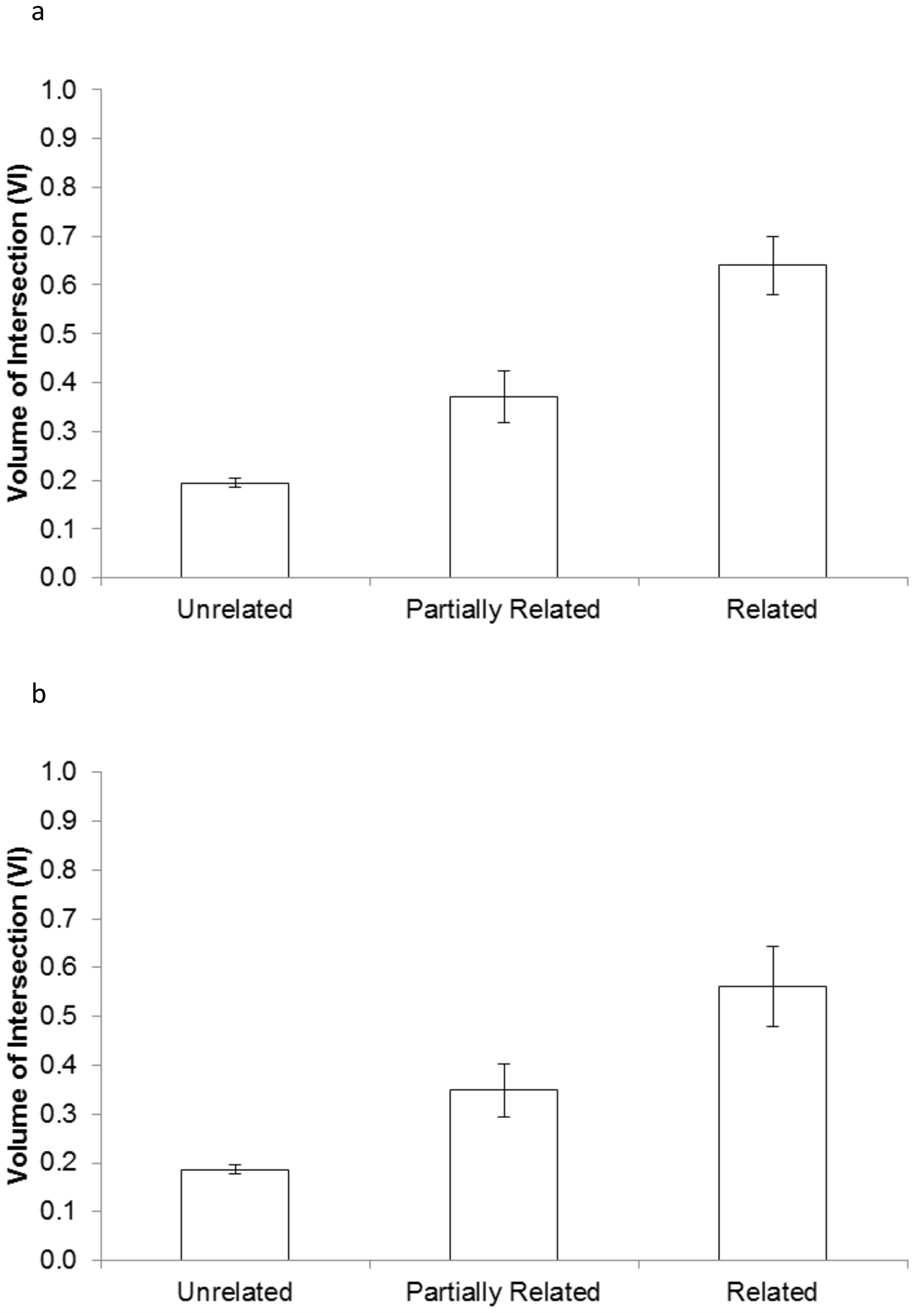
Charts detailing the average degree of overlap among deer in different categories of relatedness. Unrelated indicates R_xy_ values between 0 and 0.25, partially related indicates R_xy_ values between 0.26 and 0.5, and related indicates R_xy_ values above 0.5. Figure 2a is generated from the dataset where each pair contains at least one adult (dataset_adult_), and figure 2b is generated from the dataset using only deer-pairs from capture groups 1 and 2 (dataset_capgroup_). Figure 2a. Figure 2b.

Deer relatedness (R_xy_) had a positive association with spatial overlap (Figure 1, 2); however, this association had poor explanatory power (R^2^<0.10). Based on AIC values, the best models were those containing both kinship categories and age classes. The second best model for dataset_adult_ contained kinship categories and age-sex classes that differed for male and female fawns. This model has lower support from the data, as it was separated by 1.95 AIC units, with 27% of the total model weight compared to 71% for the top model. All other models were separated by at least 7.6 AIC units with <2% of the total model weight indicating virtually no support. For dataset_capgroup_, all other models were separated from the top model by >4.69 AIC units, with <8% of the total model weight, indicating very low support from the data.

For adult deer (dataset_adult_), first order kin had significantly higher spatial overlap (Odds Ratio = 32.46+95% CI: 5.14–204.87) than unrelated deer (Table 2). Second order kin also had much higher overlap than unrelated deer (OR = 5.42, CI: 1.17–25.00). Adult females also had higher spatial overlap with fawns than with other adults (OR = 3.06, CI: 1.35–6.98) or than with yearlings, but adult-yearling pairs were not significantly different (*p*>0.05) from adult female pairs (Table 2). For dataset_capgroup_ (all ages), first (OR = 47.47, CI: 4.26–528.90) and second (OR = 5.16, CI: 0.68–38.81) order kin again had significantly higher overlap than unrelated deer (Table 2). Among the different age classes, fawn-fawn pairs had significantly higher overlap than did adult females (OR = 11.36, CI: 1.60–80.64). In addition, adult-fawn pairs tended to have higher spatial overlap than adult-yearling or yearling-yearling pairs (Table 2).

**Table 2:** Parameter estimates from top models used to predict white-tailed deer spatial overlap in Wisconsin.

Dataset	Variable	Estimate	SE	Odds Ratio	Odds Ratio Lower 95% CI	Odds Ratio Upper 95% CI
Adult	Intercept	−2.76	0.55			
	Second Order Kin	1.69	0.78	5.42	1.17	25.00
	First Order Kin	3.48	0.94	32.46	5.14	204.87
	Age- AF	1.12	0.42	3.06	1.35	6.98
	Age- AY	−0.72	0.38	0.49	0.23	1.03
Capgroup	Intercept	−3.19	1.01			
	Second Order Kin	1.64	1.03	5.16	0.68	38.81
	First Order Kin	3.86	1.23	47.47	4.26	528.90
	Age- AF	0.88	0.64	2.41	0.69	8.45
	Age- AY	−1.05	0.55	0.35	0.12	1.03
	Age- YY	−1.05	0.74	0.35	0.08	1.49
	Age- YF	0.20	0.68	1.22	0.32	4.63
	Age- FF	2.43	1.00	11.36	1.60	80.64

Dataset_adult_ contains only deer-pairs including at least one adult deer, and dataset_capgroup_ contains only capture groups 1 and 2 (see text). Estimates for first order (R_xy_>0.5) and second order kin (0.5> R_xy_>0.25), and resultant odds ratios, are with respect to unrelated deer (R_xy_ <0.25). Estimates for age group pairs, and resultant odds ratios, are with respect to pairs consisting of two adults. In age group pairs A = adult, Y = yearling, F = fawn.

## Discussion

Social interactions, as well as group membership, may influence transmission of wildlife diseases [1], [2] and relatedness may be more important for transmission than simple proximity [21]. However, proximity can be a poor surrogate for relatedness [14] or group membership [6]. While related female white-tailed deer form social clusters on the landscape [9], [21], [44], social groups may overlap in space, but not in time. Thus, proximity of deer alone is not enough to discern relatedness, and by extension, the likelihood of transmission of infectious diseases [21]. Even adult females and fawns trapped in the same location are not always mother-offspring pairs [45]. The mechanisms by which related deer transmit infectious disease to one another are unclear, however. Because volume of intersection is a useful predictor of both direct and indirect contact rates in deer [6], it appears that related deer are more likely to come into contact, and therefore drive the dynamics of infectious diseases [21]. We identified a clear relationship between overlap (as measured by a volume of intersection) and relatedness for white-tailed deer in south-central Wisconsin. In addition, we found that age of deer influenced degree of overlap, with adult-fawn, yearling-fawn, and fawn-fawn pairs overlapping more strongly, whereas adult-adult pairs, and adult-yearling and yearling-yearling pairs exhibited lower overlap. Kinship categories were stronger predictors than continuous Rxy values, suggesting that deer beyond a certain degree of relatedness exhibit higher amounts of overlap. Even within kinship classifications, such as half-siblings or parent and offspring, there is variation in the proportion of shared DNA, and thus degree of relatedness may be less important than the nature of the social relationship between individual deer (e.g., parent offspring vs. cousins).

We found that first order kin had 32.5 times as much overlap as unrelated deer. This value is somewhat larger than a previous finding that deer in Illinois had 5.0–22.1 times greater odds of direct contact when they belonged to the same social group, as estimated by proximity [6]. However, our results may be closer to a separate study in Wisconsin indicating that deer were >100 times more likely to become infected with CWD when a highly related infected female was in close proximity, with much lower effects from proximal unrelated animals [21]. This indicates that probability of CWD infection is likely higher among closely related deer, because they have much higher contact rates, as opposed to unrelated deer that simply share space, but have lower contact rates. In addition, a higher probability of transmission may occur because of the more intense nature of contacts among related deer [46], [47]. Previous observational studies indicate that parent-offspring pairs engage in significant contact during the first year of life [46]. Studies that investigate the spatial dynamics of disease transmission in wild populations should include direct observation of deer behavior to more thoroughly address the heterogeneous disease transmission that result from the social structure of deer [22].

Our finding that adult-fawn pairs had higher overlap is not surprising given patterns of maternal care in white-tailed deer [8]. The average VI of probable parent-offspring pairs (adult-fawn pairs with R_xy_ values >0.5) were very high (0.64 in dataset_adult_, 0.55 in dataset_capgroup_), compared to the overall mean (0.22 in dataset_adult_, 0.20 in dataset_capgroup_). Adult-fawn pairs with moderate relatedness value (0.26< R_xy_ <0.5) exhibited lower overlap (0.36 in dataset_adult_, 0.36 in dataset_capgroup_). Those pairs with low (R_xy_ <0.25) relatedness values had VI values approximating the overall mean (0.24 in dataset_adult_, 0.22 in dataset_capgroup_, respectively). In this system yearling females rarely disperse from their natal home range [24], [25]. However, yearling females sometimes establish home ranges on the periphery of their mother’s home range once they breed [5], which may help explain slightly reduced overlap between adult and yearling deer.

Female white-tailed deer are highly philopatric, characterized by stable home ranges with a high degree of overlap among individuals within social groups [6], [9], [44], [46]. However, social structure of deer is less typical where rates of harvest are high and age structure is biased towards young animals [14], [48]. Nonetheless, we found that overlap (as measured by VI) closely associated with degree of relatedness, providing evidence for social structure at a local scale in spite of heavy harvest pressure. While ongoing disease eradication efforts may have temporarily increased deer harvest, this population has been subjected to ongoing harvest for many years, and CWD control efforts are unlikely to have produced the patterns observed. The strong matriarchal social structure of female white-tailed deer likely prevents homogenous mixing of individuals [6], [9] and homogeneous CWD transmission among members of different social groups [21].

The degree to which deer contact each another varies seasonally [6, 56]. However, to ensure sufficient observations, our analyses were based on annual data and provide no insight into seasonal patterns. In addition to relatedness, hotspots of activity such as scrapes, rubs, feeding/baiting sites, and mineral licks also likely play a role in contact rates of cervids and potential disease transmission [49], [50], [51]. We did not identify such features in our study and have no basis to evaluate the contribution of these behavioral hotspots to potential transmission of disease. Deer may also be more likely to overlap in agricultural areas due to concentrated food sources [3], [16], [25], [52]. In fragmented systems, deer would likely congregate closely in areas of remaining resources, particularly in seasons when food is limited [52]. Unfortunately, accuracy of the spatial locations in this study was insufficient to investigate the effects of habitat use, given that the study area is a complex mosaic of forested and agricultural land [25]. Our study focused on female deer because they are most often targeted for population control and, unlike males, rarely engage in long-distance movements [9], [21], [25]. However, males are more frequently CWD positive than females (Grear et al. 2006), and long-distance movements by males may be important in the geographic spread of CWD.

CWD can be transmitted both directly (by deer-to-deer contact) and indirectly (via contamination of the environment), though the importance of these modes of transmission in the wild are unknown [17], [19], [21]. VI provides a metric for both direct and indirect contact, though the spatial-temporal resolution of our data is insufficient to differentiate these specific events. While it is possible for two deer who overlap in space to avoid direct contact, indirect contact is virtually guaranteed, particularly given the likely occurrence of congregation points such as scrapes, rubs, feeding/baiting sites, and mineral licks. However, given previous findings that contact rates vary predictably with VI [6], we believe our findings likely apply for both direct and indirect transmission scenarios. Our findings support previous research that suggest CWD should spread more rapidly among related deer [21]. As such, control of large related social groups may be an effective strategy in slowing pathogen transmission, particularly given that there is little evidence that female harvest impacts movement behavior [16]. We also found limited overlap among unrelated deer, suggesting that disease spread among social groups, which is needed to sustain disease, may occur between neighboring social groups. We believe the rate and mechanisms of disease transmission between adjacent social groups is an important area for future research.

We provide an important step in understanding the mechanisms underlying observed patterns of CWD transmission, namely, that related individuals are more likely to come into close proximity on the landscape, where disease transmission may occur either directly or indirectly. Further progress in understanding the specifics of disease spread will be necessary to devise practical strategies for deer management.
